# Functional Genetics in the Post-genomics Era: Building a Better Roadmap in *Drosophila*

**DOI:** 10.1534/g3.113.007351

**Published:** 2013-09-01

**Authors:** Rob J. Kulathinal

**Affiliations:** Department of Biology, Temple University, Philadelphia, Pennsylvania 19122

**Keywords:** UP-TORR, FlyPrimerBank, model organism database, RNAi, online resource, genetic map, genomic roadmap

## Abstract

In this commentary, Rob Kulathinal describes two papers from the Perrimon laboratory, each describing a new online resource that can assist geneticists with the design of their RNAi experiments. Hu *et al*.’s “UP-TORR: online tool for accurate and up-to-date annotation of RNAi reagents” and “FlyPrimerBank: An online database for *Drosophila melanogaster* gene expression analysis and knockdown evaluation of RNAi reagents” are published, respectively, in this month’s issue of *GENETICS* and *G3: Genes*|*Genomes*|*Genetics*.

One hundred years ago, the very first published genetic map provided researchers with a preview of the fruit fly’s genomic landscape (Sturtevant 1913). In “The linear arrangement of six sex-linked factors in Drosophila, as shown by their mode of association,” Sturtevant (1913) estimated the order and distance between each of five X-linked factors by simply enumerating the frequency of recombinants as a direct function of the number of regional crossover events. Decades later, Sturtevant commented on the rationale of this seminal paper on the basis of his undergraduate work in Morgan’s prolific Drosophila lab at Columbia University:

*…in conversation with Morgan*… *I suddenly realized that the variations in strength of linkage*, *already attributed by Morgan to differences in the spatial separation of genes*, *offered the possibility of determining sequences in the linear dimension of a chromosome. I went home and spent most of the night (to the neglect of my undergraduate homework) in producing the first chromosome map*, *which included the sex linked genes y*, *w*, *v*, *m*, *and r*, *in the order and approximately the relative spacing that they still appear on the standard maps*. (Sturtevant, pg. 47, History of Genetics 1965)

From a surveying perspective, this work is as foundational to geneticists as early cartographical principles are to geographers. Sturtevant not only provided the logical framework for charting abstract genetic factors onto a physical and visible structure (*i.e.*, the chromosome) but also generated the first survey or map for Drosophila researchers to build upon, eventually leading to the ultimate of all genetic maps, the modern genome assembly (Adams *et al.* 2000; Celniker *et al.* 2002). Subsequently, and perhaps more importantly, this genomic roadmap provided a framework to add an odd assortment of new layers of topological features in Drosophila, such as gene model annotations (Marygold *et al.* 2013), regulatory regions and expression levels (Celniker *et al.* 2009), population variation (Langley *et al.* 2012; Mackay *et al.* 2012), and an assortment of genomic resources enabling researchers to functionally interrogate this important genetic model (see Drosophila Board White Paper; Drosophila Board of Directors 2012).

One of the most popular of these interrogation devices is RNAi (RNA interference), which has been used effectively to characterize gene function by “knocking-down” or silencing the gene expression of targeted genes. Whether in cell culture or *in vivo*, a number of genome-wide reagents have been developed and made available across a number of model organisms (for reviews of RNAi screens, see Qu *et al.* 2011 and Mohr and Perrimon 2012). However, like most genome-wide reagents, the quality of the underlying genomic roadmap is critical for proper application of these reagents. The challenge is that all genomes, including the finished *Drosophila melanogaster* genome and its perpetually improving gene models, are in a constant state of flux: new time- and spatial-specific transcripts are regularly being discovered and annotated by an ever-active community. Thus, regular updates must be reconciled with RNAi reagents, including the identification of potential off-target sites. Although FlyBase does an outstanding job in integrating and updating new annotations, most smaller online resources in the database ecosystem cannot keep up and provide outdated, and often-erroneous, information. Access to the most recent integrative roadmap is especially important for RNAi reagents and the conclusions gleaned from their experiments.

A pair of new online resources aims to alleviate this problem. In the first of two articles from Norbert Perrimon’s laboratory, Hu *et al.* (2013a) introduce a new online resource called UP-TORR (*i.e.*, Updated Targets of RNAi Reagents) that enables researchers to map RNAi reagents from major collections of flies, in addition to worms, mice, and humans. With new RNAi reagents quickly being developed and gene models under frequent refinement, this publically accessible resource will be of value to a wide range of investigators trying to understand the function of their particular gene of interest. UP-TORR provides a much-needed platform that leverages daily annotation updates from model organism databases to better define the genomic sequence that particular RNAi reagents will affect, including transcript specificity. The tool is user-friendly, allowing for a variety of inputs. The output is in tabular form and provides links to RNAi reagents from their respective library databases, thus providing a promotional portal for users to find experimental tools of interest.

The second paper describes a new online resource called FlyPrimerBank that serves to help Drosophila researchers quantify endogenous gene expression levels on any protein-coding gene of interest using ready-made primers (Hu *et al.* 2013b). These primers were especially designed to evaluate RNAi-mediated gene knockdowns from a variety of public RNAi libraries. The authors offer a comprehensive and high quality set of three primer pairs for quantitative polymerase chain reaction for every protein-coding gene providing users with alternative routes to amplifying the RNAi reagent itself. The primer sequence bank is described in detail and users are encouraged to provide community feedback about their experiences with specific primers.

As the topological features of our roadmaps become richer and more nuanced, the opportunity to make novel discoveries significantly increases. Model organism databases and the development of new online resources such as UP-TORR and FlyPrimerBank will not only help facilitate our exploration of complex genomic terrains, but can also transform our scientific approach. With a growing volume of genome-wide information at our fingertips, experiments can be better designed by integrating other functional information beforehand, as opposed to the typical *post hoc* analysis approach. With the use of genome-wide priors amassed from previously developed tools, reagents, and resources, such an informed approach will significantly reduce the time and money involved in running simple functional experiments and, once again, point to the practical importance of model system genomics. One could only imagine the reaction of the Morgan laboratory if they could see the richness and power of today’s genomic roadmap that they initially surveyed a century ago.
